# The application of 5E rehabilitation management mode in the nursing of patients with aortic dissection complicated by obstructive sleep apnea

**DOI:** 10.1007/s11325-023-02977-z

**Published:** 2023-12-29

**Authors:** Li-ling Huang, Yi Yang, Jin-hua Guo, Yi-lin Huang, Li-xia Lin

**Affiliations:** 1 Department of Cardiology, Guangdong Provincial People’s Hospital (Guangdong Academy of Medical Sciences), Guangzhou, 510080 China; 2 Department of Rehabilitation, Guangdong Provincial People’s Hospital (Guangdong Academy of Medical Sciences), Guangzhou, 510080 China

**Keywords:** 5E rehabilitation management mode, Informatization, Continuous nursing, Aortic dissection, Obstructive sleep apnea

## Abstract

**Objective:**

This study was designed to explore the effect of 5E rehabilitation mode (encouragement, education, exercise, employment, and evaluation) in patients with aortic dissection (AD) complicated by obstructive sleep apnea (OSA).

**Methods:**

Patients with Stanford type B AD (TBAD) complicated by OSA were admitted to Guangdong Provincial People’s Hospital from January 2019 to December 2020. They were randomly divided into an experimental group and a control group. After discharge, patients in the control group were given routine nursing and follow-up education, whereas patients in the experimental group were given 5E rehabilitation management mode–based nursing and follow-up education. Upon the nursing intervention, the differences in polysomnography (PSG) parameters, medication adherence, quality of life, blood pressure, and heart rate of patients between the two groups were compared. Logistic regression analysis was performed to evaluate the risk factors for the occurrence of adverse aortic events.

**Results:**

A total of 89 patients were enrolled, 49 in the experimental group and 40 in the control group. After the intervention, the control of heart rate, systolic blood pressure, medication adherence, PSG parameters, and quality of life scores in the experimental group were significantly better than those in the control group (*P*<0.05). The incidence of adverse aortic events including aortic rupture and progressive aortic dilation in the experimental group was significantly lower than that in the control group (*P* < 0.05). Logistic regression analysis revealed that acute TBAD [odds ratio (OR) = 15.069; 95%confidence interval (CI), 1.738–130.652; *P*=0.014], history of chronic kidney disease (OR=10.342; 95%CI, 1.056–101.287; *P*=0.045), and apnea hypopnea index (AHI) ≥ 30 (OR=2.880; 95%CI, 1.081–9.51; *P*=0.036) were adverse factors affecting adverse aortic events; while 5E rehabilitation management mode (OR=0.063; 95%CI, 0.008–0.513; *P*=0.010) was a favorable factor for occurrence of adverse aortic events.

**Conclusion:**

The findings suggest that continuous nursing based on information carrier 5E rehabilitation management significantly enhanced medication adherence, improved patients’ overall quality of life, and decreased the incidence of adverse aortic events in patients TBAD patients and OSA.

## Introduction

Cardiovascular diseases are now the leading cause of death. Aortic dissection (AD) is a cardiovascular disease with high mortality. Obstructive sleep apnea (OSA) is closely related to many cardiovascular diseases, including hypertension, heart failure, coronary artery disease, and atrial fibrillation [1, 2]. The main clinical manifestations of OSA consist of snoring, apneas, disorders of sleep structures, and recurrent intermittent hypoxemia [3]. Patients with OSA may develop a series of pathological disorders due to sudden narrowing or complete closure of the upper airway, which will have adverse effects on the cardiovascular system [4]. Studies have revealed the close relationship between AD and OSA [5]. OSA may promote the occurrence of AD or lead to poor prognosis of patients with AD by affecting blood pressure. The challenge of stable blood pressure control in AD is significantly compounded by the potential effects of OSA, which include blood pressure rhythm abnormality, nocturnal blood pressure elevation, and diastolic blood pressure at first awakening [6]. One study revealed that the prevalence of nocturnal apnea hypopnea index (AHI) higher than 5 and 15 in patients with AD is 66.2% and 29.1%, respectively [7]. Therefore, comprehensive management is needed to maintain the stability of blood pressure for AD in patients with OSA. At present, the post-discharge follow-up mode for such population in China is in the developmental stage. The Life Options Rehabilitation Advisory Council (LORAC) proposed a 5E mode, namely encouragement, education, exercise, employment, and evaluation [8]. Zhao et al. claimed that the 5E model could improve dietary salt control and reduce hospitalizations in patients with stable chronic heart failure [9]. Wang et al. also demonstrated that the 5E model could enhance the self-management ability of elderly patients with coronary heart disease after percutaneous coronary intervention (PCI), improve their knowledge of the disease, reduce the recurrence, and cut down the readmission rate [10]. The above studies have indicated that the 5E model significantly improved the rehabilitation and treatment outcomes of patients with cardiovascular disease after discharge from the hospital. Therefore, in this study, 5E mode-based continuous nursing combined with a follow-up model was applied in the treatment of patients with Stanford type B AD (TBAD) complicated by OSA. 

## Data and methods

### General information

Patients with TBAD complicated with OSA diagnosed in the Department of Cardiology, Guangdong Provincial People’s Hospital, from January 2019 to December 2020 were enrolled in this study. The inclusion criteria were shown as follows: (1) the diagnostic criteria of TBAD was in compliance with 2014 ESC Guidelines on the diagnosis and treatment of aortic diseases [11], (2) subjects had clear mind, with basic ability to understand and communicate; (3) patients were diagnosed with OSA; (4) participants had stable medical histories regarding other chronic conditions; (5) specific age and gender criteria were applied to define the target demographic, ensuring homogeneity within the participant group; (6) all participants provided informed consent.

Exclusion criteria included patients (1) who are unconscious, delirious, and unable to complete the questionnaire; (2) with other serious complications or unstable conditions or death during hospitalization; and (3) unwilling to participate in this study. The patients enrolled were randomly divided into an experimental group and a control group using a random number table. This study was approved by the Ethics Committee of Guangdong Provincial People’s Hospital (2020-046H-1).

### Methods

Patients in the control group were given routine nursing and follow-up education after discharge. The follow-up education included medication, diet, exercise, family blood pressure self-monitoring, patient health education, and referral education. The 5E mode was applied in the nursing of patients in the experimental group for 3 months of intervention [12]. Based on five aspects of encouragement, education, exercise, employment, and evaluation, the nursing and follow-up management from admission to discharge to long-term follow-up were designed according to the psychological state, self-management level, and influencing factors of patients in the early stage. The final outcome was evaluated 3 months after discharge.

The 5E mode management includes the following specific contents.

1. Encourage patients

Full encouragement and support were given to patients from admission to discharge. Notably, the mutual support, encouragement, companionship, and supervision among patients and their family members were highlighted.

(2) Education after discharge

Targeted education was carried out for patients. Cardiac rehabilitation guidance from doctors was issued, and related literature research was performed to develop a TBAD patient family sports manual [13]. Besides, one-to-one education was conducted during hospitalization. Communication with patients was achieved through lectures, peer education, QQ group and WeChat public platform, and other ways. After discharge, the management of blood pressure and heart rate was on the agenda. Furthermore, some interesting courses on health knowledge of OSA and experience of OSA complications were established.

(3) Exercise guidance

Exercise guidance continued until the patient returned to the family and society. The exercise mode, intensity, frequency, timing, and duration of each exercise for each patient was designed based on their own physical condition and the assessment of the rehabilitation therapist. The selected exercise mode mainly depended on the disease status. To ensure the stability of blood pressure and heart rate of the patient, active exercise in bed was carried out based on evidence-based evidence, including abdominal breathing, upper limb, and lower limb activities, active flexion of bilateral shoulder joints, abduction and inward retraction, active flexion and extension of ankle joints, and bedside and slow walking in the ward. During these exercises, the blood pressure (130–150 mmHg/80–90 mmHg) and heart rate (less than 100 times/min) of patients with dissection were kept within the safe range [14]. Changes in blood pressure, heart rate, and sensation needed to be observed and recorded before and after exercise, and it was necessary to communicate with doctors and nurses in time to reasonably adjust the exercise prescription of patients. After discharge, the patients were provided with follow-up through WeChat group or QQ group and telephone, regular consultation and communication of sports and physical status, and continuous exercise guidance.

(4) Employment

Through follow-up, the physical conditions of patients were assessed. If the condition permits, the patients would be encouraged to actively participate in social activities.

(5) Assessment

The health follow-up records of patients with TBAD and OSA were established, including personal basic information, health knowledge, and self-management assessment.

### Follow-up records

Follow-up records were routinely made once per month, and those with serious illness were followed up once per half month. Follow-up records included methods, dates, patient symptoms, and general conditions. Follow-up plans were formulated according to the severity of the patient’s condition. Telephone follow-up, SMS, WeChat, outpatient follow-up, and other services were carried out to master the patient’s diet, exercise, medication, and other compliance. The treatment plans and intervention measures were adjusted in a timely manner to ensure that medical and nursing services could be extended.

### Baseline data

General demographic data of the subjects were collected, including age, gender, educational level, economic status, length of hospital stay, blood pressure, and heart rate.

### Evaluation of polysomnography (PSG) parameters

Polysomnography (PSG) measurements were performed before and after the intervention, including AHI (events/h), sleep latency (min), total sleep time (min), sleep efficiency (%), stage 1 sleep (%), rapid eye movement (REM) sleep (%), wake after sleep onset (WASO, min), total arousal index (events/h), respiratory arousal index (events/h), minimum oxygen saturation (SpO_2_, %), and hypopnea index (events/h).

### Assessment of medication adherence

The Morisky Medication Adherence Scale is a four-item scale designed in 1986 for the assessment of medication adherence (Morisky Questionnaire) [15]. The four items are displayed as follows: (1) Do you have the experience of forgetting medication? (2) Do you sometimes not pay attention to medication? (3) When you consciously improve symptoms, have you stopped medication? (4) Have you stopped taking medicine when your symptoms are worse? Answering of yes was scored 0, of no scored 1. The higher score indicated better medication adherence.

### Quality of life assessment

The simplified 36-Item Short Form Health Survey (SF-36) consists of 12 items in eight dimensions, namely physical function, role physical, bodily pain, general health, vitality, social function, role emotional, and mental health. The scale is scored on a percentage basis. The obtained rough score was converted by the standard scoring method. The higher the score, the better the quality of life. The total Cronbach *α* coefficient of the scale was 0.775.

### Assessment of adverse aortic events

Adverse aortic events included aortic rupture, surgery or endovascular aortic repair, and progressive aortic dilation [16]. The incidence of these events was assessed before and after intervention.

### Statistical analysis

The data were input into EpiDate software and compared and then imported into SPSS22.0 software. Continuous variables with normal distribution were expressed as mean ± standard deviation (SD), and a *t*-test was used to compare the differences between the two groups. Categorical variables were presented as counts or percentages, and differences between the two groups were analyzed using the chi-square test or Fisher’s exact test. In addition, logistic regression analysis was employed to assess the independent risk factors of the occurrence of adverse aortic events. *P* < 0.05 was considered a statistically significant difference.

## Results

### Baseline data of two groups

A total of 89 patients were included in this study. There were 40 patients (33 men) in the control group and 49 in the experimental group (45 men). As shown in Table 1, no significant difference was exhibited between the two groups in terms of gender ratio, age, body mass index (BMI), education level, history of hypertension, dyslipidemia, history of diabetes, smoking history, chronic kidney disease, and acute TBAD (*P* > 0.05).

**Table 1:** Comparison of baseline data between the experimental group and control group

	Control group (*n* = 40)	Experimental group (*n* =49)	Statistic	*P*
Age	53.4 ± 10.9	57.4 ± 11.0	−1.721	0.089
Gender, male/female	33/7	45/4	1.015	0.314
BMI	24.2 ± 3.7	24.90 ± 3.7	−0.955	0.342
Education level			5.429	0.143
Primary school	12	8		
Junior high school	15	29		
High school	10	7		
College degree or above	3	5		
History of hypertension	17 (42.5)	15 (30.6)	1.352	0.245
Dyslipidemia	9 (22.5)	12 (24.5)	0.048	0.826
History of diabetes	8 (20.0)	9 (18.4)	0.038	0.845
Smoking history	3 (7.5)	4 (8.2)	0.013	0.908
Chronic kidney disease	11 (27.5)	13 (26.5)	0.011	0.918
Acute TBAD	13 (32.5)	10 (20.4)	1.680	0.195
Aortic diameter (mm)	5.09±0.30	5.18±0.19	−1.617	0.110
History of cardiovascular diseases	13 (32.5)	13 (26.5)	0.379	0.538
Complex TBAD	12 (30.0)	11 (22.4)	0.655	0.418
PCI treatment history	5 (12.5)	3 (6.1)	1.095	0.295
History of coronary artery bypass grafting	7 (17.5)	5 (10.2)	1.005	0.316
β blocker	5 (12.5)	4 (8.2)	0.456	0.500
ACEI/ARB	7 (17.5)	6 (12.2)	0.488	0.485
Calcium antagonists	7 (17.5)	7 (14.3)	0.172	0.679

Values are mean ± SD or *n* (%). BMI, body mass index; TBAD, Stanford type B aortic dissection; *PCI*, percutaneous coronary intervention; *ACEI*, angiotensin-converting enzyme inhibitor; *ARB*, angiotensin receptor blocker

### Changes in blood pressure and heart rate of patients before and after intervention

Before the intervention, there was no significant difference in systolic blood pressure, diastolic blood pressure, and heart rate between the two groups (*P* > 0.05). After the intervention, the systolic blood pressure in the experimental group was better than that in the control group (*P* < 0.001). Besides, the average heart rate was 77.8 ± 10.9 times/min in the experimental group and 74.3 ± 10.7 times/min in the control group before the intervention. But after the intervention, it changed to 68.8 ± 5.1 times/min in the experimental group and 70.0 ± 8.0 times/min in the control group (Table 2). Such results indicated that the control of heart rate in the experimental group was much better than that in the control group.

**Table 2:** Changes in blood pressure and heart rate of patients before and after 5E mode intervention

	Before intervention		*P*	After intervention		*P*
	Experiment group	Control group		Experiment group	Control group	
Systolic pressure (mmHg)	129.8±9.2	130.2±9.6	0.848	115.4±2.7	121.4±3.2	< 0.001
Diastolic pressure (mmHg)	79.1±12.9	78.5±13.6	0.812	65.6±3.3	65.5±3.9	0.954
Heart rate (times/min)	77.8±10.9	74.3±10.7	0.135	68.8±5.1	70.0±8.0	0.407

Values are mean ± SD

### Changes in PSG parameters before and after 5E mode intervention

The effect of the 5E mode intervention on PSG parameters was analyzed in both control and experimental groups (Table 3). Compared with the pre-intervention period, patients in the control group exhibited much lower WASO and respiratory arousal index after intervention (*P* < 0.001). Before the intervention, AHI, sleep latency, WASO, respiratory arousal index, and hypopnea index were significantly lower in the experimental group than in the control group, while sleep efficiency, the percentage of REM, and minimum SpO_2_ were significantly increased (*P* < 0.001). After the intervention, AHI, sleep latency, WASO, respiratory arousal index, and hypopnea index of the experimental group were significantly lower than those of the control group (*P* < 0.001), while sleep efficiency, minimum SpO_2_, and percentage of REM were significantly higher (*P* < 0.001). However, there were no significant changes in stage 1 sleep, total arousal index, and total sleep time between the two groups before and after the intervention (*P* > 0.05).

**Table 3:** Changes in polysomnography parameters of the two groups of patients before and after 5E mode intervention

	Before intervention	After intervention	*t*	*P*
AHI, events/h				
Control group	7.4±2.4	7.5±2.5	−0.595	0.555
Experimental group	6.9±2.1	5.0±2.0	14.412	< 0.001
*t*	0.999	5.237		
*P*	0.321	< 0.001		
Sleep latency, min				
Control group	14.8±3.2	15.7±2.7	−1.342	0.187
Experimental group	14.5±2.7	11.2±3.3	6.522	< 0.001
*t*	0.480	6.913		
*P*	0.632	< 0.001		
Total sleep time, min				
Control group	332.5±21.4	332.8±21.0	−0.502	0.618
Experimental group	333.3±20.6	336.8±24.8	−1.327	0.191
*t*	−0.171	−0.812		
*P*	0.864	0.419		
Sleep efficiency, %				
Control group	78.7±5.8	78.8±5.7	−0.697	0.490
Experimental group	78.2±6.7	84.8±6.7	−54.067	< 0.001
*t*	0.397	−4.486		
*P*	0.692	< 0.001		
Stage 1 sleep, %				
Control group	83.0±2.3	82.6±2.7	1.267	0.213
Experimental group	83.1±2.2	83.2±3.01	0.375	0.710
*t*	−0.269	−1.026		
*P*	0.789	0.308		
REM percentage, %				
Control group	19.2±3.4	18.6±3.3	2.009	0.051
Experimental group	17.9±3.7	22.7±2.6	−9.806	< 0.001
*t*	1.580	−6.522		
*P*	0.118	< 0.001		
WASO, min				
Control group	54.3±11.7	49.8±11.9	29.002	< 0.001
Experimental group	51.6±9.7	43.8±9.9	36.349	< 0.001
*t*	1.191	2.524		
*P*	0.237	0.014		
Total arousal index, events/h				
Control group	11.0±2.3	11.6±2.1	−1.332	0.191
Experimental group	11.5±2.5	11.5±2.3	0.042	0.966
*t*	−1.167	0.095		
*P*	0.246	0.924		
Respiratory arousal index, events/h				
Control group	6.7±1.8	6.5±1.7	0.507	0.615
Experimental group	6.8±1.5	4.3±1.6	34.790	< 0.001
*t*	−0.217	6.456		
*P*	0.829	< 0.001		
Minimum SpO_2_ (%)				
Control group	89.2±2.7	89.4±2.8	−1.481	0.147
Experimental group	89.1±2.5	92.7±2.5	−20.279	< 0.001
*t*	0.149	−5.772		
*P*	0.882	< 0.001		
Hypopnea index, events/h				
Control group	8.4±2.3	8.3±2.5	0.781	0.440
Experimental group	8.2±2.2	5.3±2.4	19.883	< 0.001
*t*	0.407	5.701		
*P*	0.685	< 0.001		

Values are mean ± SD. *AHI*, apnea hypopnea index; *REM*, rapid eye movement; *WASO*, wake after sleep onset, *SpO*_*2*_, oxygen saturation

### Changes in medication adherence and quality of life of patients before and after intervention

After the intervention, the medication adherence in the both groups was notably higher than that before the intervention (*P* < 0.001). However, there was no difference between the two groups after the intervention (Table 4). Before the intervention, there was no significant difference in the scores of the dimensions of the quality of life score between the two groups (*P* > 0.05). After the intervention, physiological function, bodily pain, general health, vitality, role emotional, and mental health scores of the patients in the experimental group were significantly higher than those of the patients in the control group (*P* < 0.05).

**Table 4:** Changes in medication adherence and quality of life of patients before and after 5E mode intervention

	Before intervention	After intervention	*t*	*P*
Medication adherence				
Control group	1.0±0.6	1.7±0.5	5.365	<0.001
Experimental group	0.9±0.6	2.1±1.3	5.639	<0.001
*t*	0.744	0.066		
*P*	0.459	1.861		
SF-36 scores				
Physiological function				
Control group	41.5±20.0	49.33±20.7	−28.479	<0.001
Experimental group	40.6±19.2	58.9±19.5	−61.193	<0.001
*t*	0.213	−2.237		
*P*	0.832	0.028		
Role physiological				
Control group	29.4±21.1	32.5±17.2	−1.403	0.168
Experimental group	31.6±19.6	33.2±21.3	−0.771	0.444
*t*	−0.522	−0.159		
*P*	0.603	0.874		
Bodily pain				
Control group	56.7±12.7	59.0±11.6	−1.906	0.064
Experimental group	53.7±14.2	69.4±14.3	−43.372	<0.001
*t*	1.064	−3.723		
*P*	0.290	<0.001		
General health				
Control group	48.9±6.2	56.9±7.1	−26.695	<0.001
Experimental group	50.6±7.6	66.2±7.9	−51.147	<0.001
*t*	−1.177	−5.825		
*P*	0.242	<0.001		
Vitality				
Control group	47.0±11.7	55.2±11.1	−23.920	<0.001
Experimental group	46.8±8.5	59.4±8.6	−34.127	<0.001
*t*	0.076	−2.037		
*P*	0.939	0.045		
Social function				
Control group	55.0±12.6	63.3±12.6	−26.300	<0.001
Experimental group	53.6±13.5	66.2±13.4	−43.862	<0.001
*t*	0.512	−1.047		
*P*	0.610	0.298		
Role emotional				
Control group	27.5±26.0	42.5±29.2	−2.682	0.011
Experimental group	23.8±22.6	57.8±21.3	−8.367	<0.001
*t*	0.716	−2.771		
*P*	0.476	0.007		
Mental health				
Control group	50.3±13.1	52.1±12.6	−1.183	0.244
Experimental group	48.7±11.2	62.8±11.3	−47.365	<0.001
*t*	0.606	−4.244		
*P*	0.546	<0.001		

Values are mean ± SD

### The impact of 5E mode intervention on the incidence of adverse aortic events in TBAD patients with OSA

There were 17 cases (42.5%) of adverse aortic events in the control group, including aortic rupture in 5 cases, surgery or endovascular aortic repair in 5 cases, and progressive aortic dilation in 7 cases. The incidence of these adverse events in the experimental group was significantly decreased to 6 cases (12.2%) (*P* = 0.001), indicating a substantial reduction in aortic complications following the implementation of the 5E mode intervention (Table 5).

**Table 5:** The impact of 5E mode intervention on the incidence of aortic related adverse events in TBAD patients with OSA

	Control group (*n* = 40)	Experimental group (*n* =49)	Statistics	*P*
Cases of deaths	0	0	-	-
Aortic rupture	5	2	-	-
Surgery or endovascular aortic repair	5	2	-	-
Progressive aortic dilation	7	2	-	-
Total incidence rate (%)	17 (42.5)	6 (12.2)	10.519	0.001

### Logistic regression analysis of the occurrence of adverse aortic events in TBAD Patients with OSA

Univariate regression analysis revealed ten indicators associated with the occurrence of adverse aortic events in patients with TBAD and OSA. These indicators included age, hypertension, complex TBAD, acute TBAD, history of chronic kidney disease, AHI ≥ 30, WASO, percentage of REM, systolic blood pressure, and the 5E rehabilitation management mode. The results of multivariate regression analysis showed that acute TBAD [odds ratio (OR) = 15.069; 95% confidence interval (CI), 1.738–130.652; *P* = 0.014], history of chronic kidney disease (OR = 10.342; 95% CI, 1.056–101.287; *P* = 0.045), and AHI ≥ 30 (OR = 2.880; 95% CI, 1.081–9.51; *P* = 0.036) were adverse factors affecting adverse aortic events, and 5E rehabilitation management mode (OR = 0.063; 95% CI, 0.008–0.513; *P* = 0.010) was a favorable factor affecting adverse aortic events (Table 6).

**Table 6:** Logistic regression analysis of adverse aortic events in TBAD patients with OSA

	Univariate regression analysis		Multivariate regression analysis	
	*OR (95% CI)*	*P*	*OR (95% CI)*	*P*
Age	0.991 (0.949–1.035)	0.678	-	-
History of hypertension	5.404 (1.949–14.989)	0.001	2.753 (0.441–17.17)	0.278
Complex TBAD	7.80 (2.512–21.000)	< 0.001	3.489 (0.506–24.043)	0.204
Acute TBAD	43.920 (11.426–168.821)	< 0.001	15.069 (1.738–130.652)	0.014
History of cardiovascular diseases	2.404 (0.886–6.523)	0.085	-	-
History of chronic kidney disease	8.711 (2.975–25.504)	< 0.001	10.342 (1.056–101.287)	0.045
AHI ≥ 30, events/h	9.964 (2.681–37.034)	0.001	2.880 (1.081–9.51)	0.036
WASO, min	1.102 (1.050–1.156)	< 0.001	1.067 (0.959–1.186)	0.234
REM percentage, %	1.244 (1.056–1.465)	0.009	1.289 (0.934–1.779)	0.122
Minimum SpO_2_, %	1.094 (0.906–1.320)	0.352	-	-
Systolic pressure	1.008 (0.981–1.037)	0.560	-	-
5E rehabilitation management mode	0.189 (0.065–0.545)	0.002	0.063 (0.008–0.513)	0.010

*OR*, odds ratio; *CI*, confidence interval; *OSA*, obstructive sleep apnea; *TBAD*, Stanford type B aortic dissection; *AHI*, apnea hypopnea index; *REM*, rapid eye movement; *WASO*, wake after sleep onset, *SpO*_*2*_, oxygen saturation

## Discussion

The 5E rehabilitation management mode–based nursing had many advantages in the management of TBAD combined with OSA. First, it significantly improved the quality of life of the patients in this study. We were able to communicate successfully with patients via the platforms QQ and WeChat as well as phone calls and other online channels. In the era of Internet plus, the 5E management mode provided a continuous, multi-form follow-up model for patients with TBAD and OSA.

This study showed that the medication adherence of patients in the experimental group after 5E mode intervention was markedly higher than that of the control group. The 5E management model is designed to foster strong bonds among patients, between patients and their families, and health care workers [17]. For medical staff, close contact with patients is needed to improve and strengthen patient compliance behavior through timely encouragement, follow-up, and guidance. 

The 5E intervention in patients with TBAD and OSA showed a significant reduction in AHI and improvement in sleep efficiency reflected in enhanced sleep quality. There was a decrease in adverse aortic events, such as aortic rupture and progressive aortic dilation, highlighting the potential of the 5E intervention in mitigating serious cardiovascular complications in this high-risk population. 

The 5E intervention also resulted in effective control of patients’ blood pressure, particularly systolic blood pressure, and heart rate [18, 19]. The effective control of blood pressure and heart rate, the treatment of risk factors, and the treatment of related atherosclerotic diseases are of importance in the development and prognosis of AD [20]. 

 Though factors like complex TBAD and hypertension history heightened risks, the 5E mode served as a robust protective factor. Such outcomes align with previous research showcasing the effectiveness of multifaceted interventions [10, 21]. 

There are limitations with this study. First, the small sample size does limit the generalizability of the findings. Additionally, the short follow-up duration restricts the ability to assess the long-term efficacy and sustainability of the 5E rehabilitation management mode. Although we focused on blood pressure and heart rate control, other essential parameters may influence TBAD and OSA outcomes. 

## Conclusion

The findings of this study suggest that the 5E rehabilitation management mode–based nursing may significantly enhance medication adherence, regulate blood pressure and heart rate, and elevate the quality of life for patients with TBAD and OSA. By integrating information-based nursing and the 5E management mode, this study not only supports the chronic management of TBAD and compliance in patients with OSA, it also lays a foundation for future chronic disease management. 

## Data Availability

The data used to support the findings of this study are available from the corresponding author upon request.
